# Optically Transparent Flexible Broadband Metamaterial Absorber Based on Topology Optimization Design

**DOI:** 10.3390/mi12111419

**Published:** 2021-11-18

**Authors:** Pingping Min, Zicheng Song, Lei Yang, Victor G. Ralchenko, Jiaqi Zhu

**Affiliations:** 1 Center for Composite Materials and Structures, Harbin Institute of Technology, Harbin 150080, China; minpp@hit.edu.cn (P.M.); songzc@hit.edu.cn (Z.S.); vg_ralchenko@mail.ru (V.G.R.); 2 Research Center of Analysis and Measurement, Harbin Institute of Technology, Harbin 150080, China; yanglei8399@163.com; 3 Prokhorov General Physics Institute of Russian Academy of Sciences, Vavilov Str. 38, 119991 Moscow, Russia

**Keywords:** broadband metamaterial absorber, optically transparent, flexible, topology optimization

## Abstract

A conformal metamaterial absorber with simultaneous optical transparency and broadband absorption is proposed in this paper. The absorptance above 90% over a wide frequency range of 5.3–15 GHz can be achieved through topology optimization combined with a genetic algorithm (GA). The broadband absorption can be kept at incident angles within 45° and 70° for TE mode and TM mode, respectively. In the meantime, by employing transparent substrates, including polyvinyl chloride (PVC) and polyethylene terephthalate (PET), good optical transmittance and flexibility can be obtained simultaneously. The experimental results agree well with the numerical simulations, which further validates the reliability of our design and theoretical analysis. With its visible-wavelength transparency, flexibility, broadband absorption, low profile, excellent angle stability and polarization insensitivity, the proposed absorber is highly favored for practical applications in microwave engineering, such as electromagnetic interference and stealth technology. Moreover, the proposed design method of topology optimization can be extended to design the absorber quickly and efficiently, according to specific engineering requirements.

## 1. Introduction

Microwave absorbers are widely used in various fields, ranging from military equipment to civil buildings and wireless communication facilities, owing to their effectiveness at dissipating microwave energy [1,2,3]. Most applications have broadband absorption requirements, such as solar photovoltaics [4], photodetection [5], the manipulation of mechanical resonances [6] and the stealth design of warship and aircraft [7,8], which makes broadband absorption an eternal pursuit for microwave absorbers. However, most of the traditional microwave absorbers, such as ferrite, conductive polymer, conductive graphite, etc., have a strong blocking effect on visible light [9,10,11]. The lack of the characteristics of optical transparency hinders their application in optically transparent absorbing devices, such as in stealth warship or aircraft window development, radio frequency identification (RFID) systems, and electronic toll collection (ETC) systems [12,13,14].

Recently, optically transparent broadband absorbers have drawn much attention [15,16,17,18,19]. Salisbury screen and Jaumann absorbers based on the transparent conductive films can achieve visible transmission and microwave absorption characteristics at the same time [20,21]. However, they have problems due to their narrow bands or large thickness, which cannot satisfy the needs of applications [22,23,24]. The introduction of metamaterials with artificial periodic structures has been a turning point for the problem, since the effective permittivity and effective permeability of metamaterials can be manipulated by designing the parameters of structures [25,26,27,28,29]. By stacking multiple layers of patterned indium tin oxid (ITO) resistive films, absorption peaks of different frequencies can be generated to overlap in the spectrum, so as to form a continuous broadband [30,31,32]. However, the multilayered structure has multiple interfaces and layers, resulting in a severe decrease in the visible light transmittance [8].

In order to improve the visible light transmittance, many studies have tried to design a transparent broadband absorber based on the sandwich structure, which is composed of a patterned impedance matching layer, an intermediate dielectric layer and a grounded reflective surface [19,33,34]. Cheng et al. [35] used polymethyl methacrylate (PMMA) as the substrate and used the windmill-shaped elements in the top layer to generate multiple resonances to obtain broadband absorption from 8.3 to 17.4 GHz. Moreover, flexibility has become an essential requirement for microwave absorbers when they are implemented for optically transparent devices [7,12,36]. Deng et al. [7] proposed a flexible transparent absorber for window application based on the lossy “wheel-like” metasurface, which can achieve 90% absorption from 6 to 16.5 GHz. Jang et al. [17] presented an optically transparent, flexible and polarization-independent broadband microwave absorber by using Al wire gird to construct the bow-tie array to induce high ohmic loss and combined resonances, which led to more than 90% total absorption, covering a wide frequency range from 5.8 to 12.2 GHz. However, the metasurface pattern of the transparent absorber based on the sandwich structure is becoming more and more complicated, so as to obtain broadband absorption, but its design method lacks versatility. The above work lacks the general paradigm and design principle of the pattern in the impedance matching layer that can achieve broadband absorption, so that it is impossible to further guide the patterned design of the sandwich structure absorber to obtain broadband absorption. Therefore, designing the absorber according to specific engineering requirements is still a huge challenge due to the lack of practical design method, especially the comprehensive performance requirements of absorbers, such as broadband absorption, optically transparency, angular stability, thinness and conformity should be fully considered.

In this study, the topology optimization method based on a genetic algorithm (GA) is proposed and applied to the fast and efficient design of the flexible transparent broadband metamaterial absorber (FTBMA). By using the combination of binary encoding and real encoding, the encoding length is shortened, which can improve the convergence speed of optimization. To verify the efficiency of the method, a conformal optical transparent metamaterial absorber with the broadband absorption ranging from 5.3 GHz to 15 GHz is designed, fabricated and characterized. High accordance between the experimental results and numerical simulations further validates the reliability of our design and theoretical analysis. Both results demonstrate excellent absorption performance of the FTBMA, which is highly favored for practical applications.

## 2. Unit Cell Design by Topology Optimization

**Figure 1:**
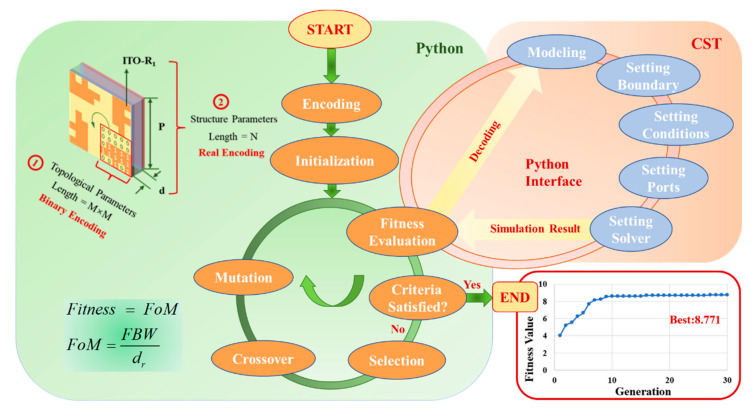
The schematic diagram of FTBMA design process by topology optimization combined with GA and the co-simulation.

The process of designing an FTBMA using topology optimization combined with GA and the co-simulation is illustrated in Figure 1. The key to the topology optimization design of an FTBMA is the encoding of metamaterials. The longer the length of the encoding, the less conducive it is to the convergence of the optimization algorithm, which means the possibility of searching for the optimal solution in a limited range becomes smaller [37,38]. Actually, in many cases, the symmetry of FTBMA structures is decided by the physical constraints and application goals. Considering the fact that FTBMAs with a stable performance under various incidence angles and different polarizations are widely desired in many fields, here we focus on the design of an FTBMA with a four-fold rotational symmetry axis to obtain polarization insensitivity.

The encoding of the FTBMA includes topological encoding and parameter encoding, as shown in the coding part in the upper left corner of the schematic diagram in Figure 1. The surface of the unit cell of the FTBMA is the optimization area of topological encoding. Since the structural unit of the FTBMA has a four-fold rotational symmetry axis, the optimization area can be further reduced to a quarter of the original area. The optimization area is divided into M × M (in this paper M = 5) small squares marked by 1 or 0. The code 1 means that the small square is filled up with the resistive patch, and the code 0 means it is filled up with air. Thus, the length of topology encoding is 25. In addition, the process of encoding the structure and the material parameters of the FTBMA, including the length of the unit cell (P), the thickness of the intermediate dielectric layer (d) and the sheet resistance of the upper ITO resistance film (R_1_) is parameter encoding. In previous reports, the parameter of the absorbers encoding method generally uses binary encoding, which usually makes the crossover operation of the GA destroy the continuous semantics of a single parameter on the chromosome, so as to reduce the optimization effect. In order to avoid this problem, real encoding is used for parameter encoding in optimization; thus, the encoding length is N (in this paper N = 3), which effectively shortens the encoding length and speeds up the optimization process. In the summary, the code length of the FTBMA is 28, as shown in Figure 1.

Full-wave numerical simulations of the FTBMA were carried out by the finite-element frequency-domain method in the CST microwave studio (CST MWS) software, while the parameters of the simulation model are provided by GA in the python software. Furthermore, in the CST MWS, floquet ports with normal incident plane transverse electric (TE) waves and transverse magnetic (TM) waves were set in the z-direction for excitation. Periodic boundary conditions (PBCs) along the x and y directions were used to simulate the infinite periodic element. The frequency-dependent complex S-parameters can be obtained by the frequency domain solver simulation. The absorptance of the FTBMA under normal incidence can be defined as A(ω) = 1 − R(ω) − T(ω), where R(ω) = |S11|^2^ and T(ω) = |S21|^2^ are the reflectance and transmittance derived from the complex S-parameter, respectively.

The optimization design of the FTBMA is a multi-objective optimization problem. In order to obtain a high visible light transmittance, the FTBMA use the metal mesh as the grounded reflective surface and adopt the sandwich structure. On this basis, the working bandwidth, absorptivity and thickness of FTBMAs should be considered at the same time when calculating the fitness function. It is worth noting that the working bandwidth of the absorber and the electrical size of the structural parameters are highly dependent. By synchronously adjusting the overall thickness of the absorber and the geometric parameters of each part, it is possible to achieve free space impedance matching and absorption at any frequency band while keeping the relative bandwidth unchanged. Generally, a fractional bandwidth (*FBW*) with an absorption rate greater than 90% is defined as the evaluation standard for absorption performance, which is expressed as follows:

(1) $$FBW=2\times \frac{f_{H}-f_{L}}{f_{H}+f_{L}},$$

where *f_H_* and *f_L_* are high and low limits of the frequency range with an absorption above a reference value (generally 90%), respectively. When there are multiple bands with absorptance above 90% within the simulated frequency band, calculate the corresponding *FBW* values respectively, and then select the maximum value of *FBW* as the reference.

Considering the absorption performance and thickness comprehensively, a figure of merit [39] [FoM = *FBW*/d_r_, d_r_ is the relative thickness of absorbers] as the fitness function is employed to evaluate the performance of the FTBMA in the process of optimization. The bigger the value of FoM, the better the performance of the FTBMA.

**Figure 2:**
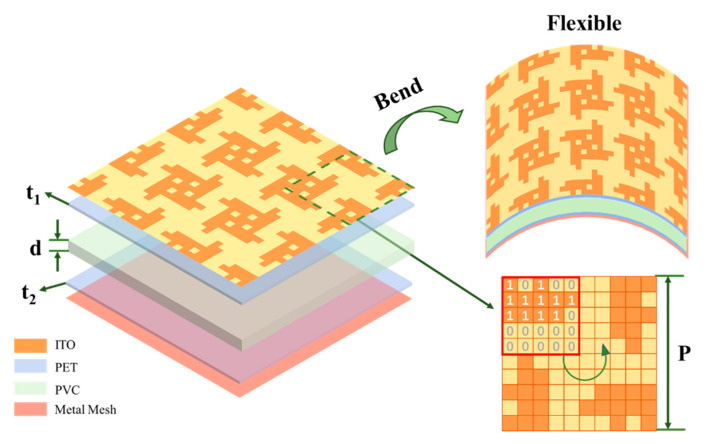
(**left**) Exploded view and (**upper right**) bending schematics of FTBMA (4 × 4 structure). (**lower right**) Top view of its unit cell configuration.

As shown in Figure 1, GA is run in the main program in python, and the CST is repeatedly called for modeling, condition setting and simulation, and finally, the calculation result is returned to the main program to form a loop until the predetermined termination condition or the design goal are reached. The optimization results were obtained by combined simulation after 30 iterative evolutions, as illustrated by the iteration curve in the lower right corner of Figure 2.

The schematic diagram of the FTBMA designed through topology optimization is shown in Figure 2. The proposed FTBMA consists of a transparent patterned ITO metasurface, a polyvinyl chloride (PVC) mat and a metal mesh backplane from the top to the bottom. PVC, whose relative permittivity ε_r_ and dielectric loss tangent are 2.4 and 0.06, respectively, is used as the intermediate dielectric layer due to its high optical transparency, ideal flexibility and good mechanical properties. Moreover, in order to improve the optical transmittance of the FTBMA, the copper metal mesh with a transmittance of 86.5% and a sheet resistance of 1 Ω/sq is used as the backplane instead of the commonly used continuous ITO with a transmittance of 74% and a sheet resistance of 6 Ω/sq. The final optimized parameters were obtained as P = 14.1 mm, d = 4 mm, R_1_ = 53 Ω/sq, as defined in Figure 2. Moreover, the thickness of the polyethylene terephthalate (PET) substrate (ε = 3.0(1 − j0.06) attached by the upper patterned ITO film and the bottom metal mesh are 0.175 mm (t_1_), 0.125 mm (t_2_), respectively.

## 3. Simulation and Analysis

**Figure 3:**
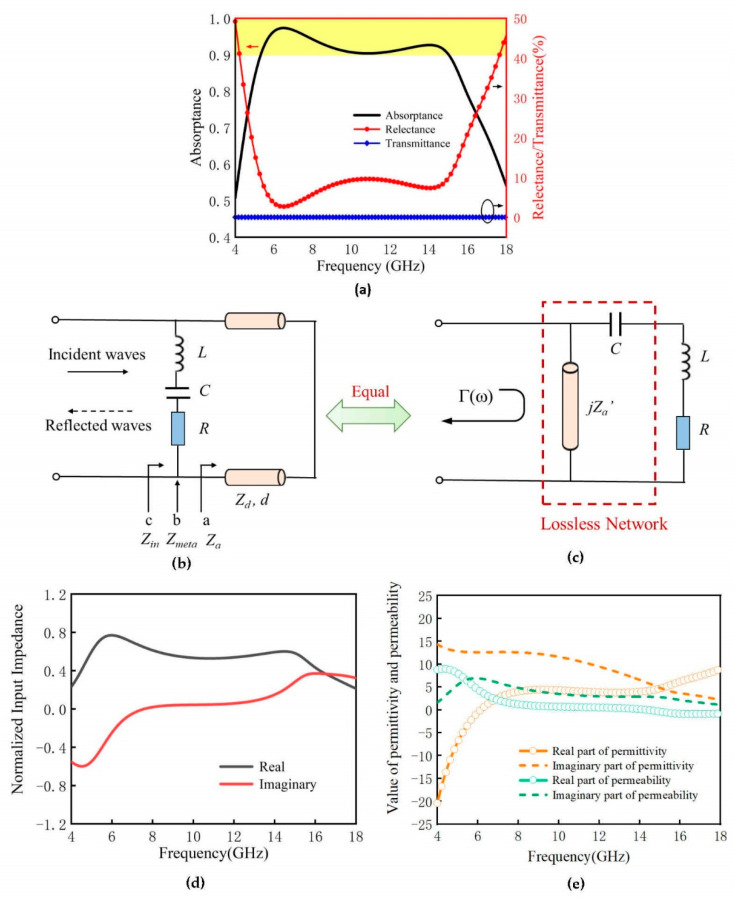
(**a**) Microwave-range characteristics of FTBMA under normal incident TE plane wave irradiation conditions. (**b**) The equivalent circuit model of FTBMA. (**c**) The circuit of series RL loads matched with passive and lossless networks. (**d**) Normalized input effective impedance of FTBMA. (**e**) Frequency-dependent spectra of retrieved effective electromagnetic parameters of FTBMA.

The proposed FTBMA can obtain broadband microwave absorption with an efficiency above 90% over the broadband of 5.3 to 15 GHz, as is apparent in Figure 3a. It should be noted that the low transmission value close to zero can be attributed to the low sheet resistance (1 Ω/sq) of the Cu-metal-mesh backplane, which may be approximated as a reflector to avoid secondary scattering.

Transmission-line theory is a powerful method to analyze the resonant behavior of an absorber. Here, a circuit model is established to give a physical insight into the absorption performance of the FTBMA. As simplification, it can be assumed that the unit cell is terminated by a shorten load, because the little loss of the Cu-metal-mesh backplane can be neglected. At the same time, the effect of PET substrate is negligible because of its ultra-thin thickness. Conforming to the response of the FTBMA in an external electromagnetic field, a general equivalent circuit of the FTBMA is established in Figure 1. The intermediate dielectric PVC layer can be treated as a fraction of the transmission line with certain characteristic impedance, while the patterned ITO metasurface on the top layer can be modeled as a series circuit composed of inductance L, capacitance C and resistance R. When the FTBMA is exposed to incident electromagnetic wave, no transmission takes place due to the Cu-metal-mesh backplane. Thus, the absorption can be written as a function of the reflectivity:

(2) $$A=1-{\left|\Gamma \right|}^{2},$$

where *A*, *Γ* are the absorptivity and reflection coefficient of FTBMA, respectively.

Looking from port ‘*a*’ towards the bottom ground plane, the transmission line impedance *Z_a_* can be derived as:

(3) $$Z_{a}=j\frac{Z_{0}}{\sqrt{\varepsilon_{r}}}\tan\frac{2\pi f\sqrt{\varepsilon_{r}}d}{c},$$

where, *ε_r_*, *d* are the relative permittivity and thickness of the PVC substrate, respectively, *f* is the frequency of the incident electromagnetic waves and *c* is the velocity of light. The transmission line impedance *Z_a_* and the equivalent capacitance C of ITO metasurface can be reasonably combined as a lossless network, because they are passive and lossless. Therefore, the equivalent circuit model of the FTBMA in Figure 3b can be reasonably equivalent to the circuit of series RL loads matched with passive and lossless networks in Figure 3c.

According to the Bode–Fano limits [40] for series RL loads matched with passive and lossless networks, the following formula can be given:

(4) $$\int_{0}^{\infty }\ln\frac{1}{\left|\Gamma \right|}d\omega =\int_{0}^{\infty }\ln\frac{1}{\sqrt{1-A}}d\omega \;=\int_{△\omega }\ln\frac{1}{\sqrt{1-A_{m}}}d\omega \;=△\omega \ln\frac{1}{\sqrt{1-A_{m}}}<\frac{\pi R}{L},$$

where *A_m_* is the passband absorptivity and *ω* is the circular frequency (*ω* = 2π*f*). It can be concluded that for a given load (a fixed R/L product), a broader bandwidth (Δ*ω*) can be achieved only at the expense of a lower absorptivity in the passband. It can be seen that there is a certain difficulty for the tradeoff between absorption quality and absorption bandwidth for the absorber design. The proposed method of designing the topology optimization based on a GA can be extended to not only the quick and efficient design of the absorber, but it can also be independently optimized and balance the relationship between absorption quality and absorption bandwidth, which has been proven by the design of the FTBMA in Figure 2.

The characteristic impedance of free space Z0 of the proposed absorber can be obtained from the plot of the normalized input effective impedance *Z_eff_*(*ω*) of the FTBMA, as calculated from the simulated *S*-parameters of the unit cell according to the effective medium theory displayed in Figure 3e via the use of Equation (5) [41]:

(5) $$Z_{eff}=\sqrt{\frac{{\left(1+S_{11}\right)}^{2}-S_{21}^{2}}{{\left(1-S_{11}\right)}^{2}-S_{21}^{2}}},$$

where *ε_eff_*(*ω*) and *μ_eff_*(*ω*) are the effective permittivity and permeability, respectively. It is obvious that the real part of the effective impedance fluctuates around 1, while the imaginary part is approximately zero in the wideband from 5.3 to 15 GHz from Figure 3e. It can be concluded that the input impedance of the FTBMA almost matches the impedance of free space Z0 in the working band.

With the help of Ziolkowski’s theory [42], *ε_eff_*(*ω*) and *μ_eff_*(*ω*) were retrieved to reveal the nature of the FTBMA. As shown in Figure 3e, both the effective permittivity and permeability vary accordingly in working bands. Metamaterial behavior that real part of effective permittivity, very close to real part of effective permeability, stays almost near to zero in the whole band of 5.3–15 GHz should also be noticed. This once again proves that the FTBMA has an excellent characteristic impedance of free space Z0 in the working band, which is beneficial to obtaining a broadband absorption for the FTBMA.

**Figure 4:**
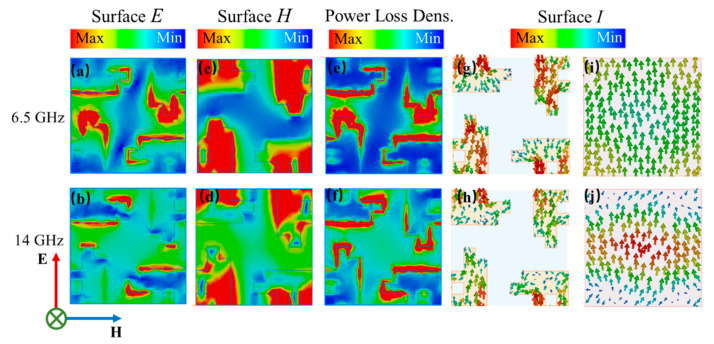
Electromagnetic responses of the proposed absorber under normally incident TE waves at 6.5 GHz and 14 GHz. The distributions of (**a**,**b**) electric field (**c**,**d**) magnetic field (**e**,**f**) power loss density. Distributions of surface currents on (**g**,**h**) the ITO resistive metasurface and (**i**,**j**) the bottom ground layer.

For insight into the physical mechanism of the broadband absorption performance of the FTBMA, surface electric field E, surface magnetic field M, surface power loss density and surface-current I distributions of the FTBMA at 6.5 GHz and 14 GHz were studied and are given in Figure 4. The surface electric field is mainly distributed in the gap of the ITO patterns, especially at the edge of the contour of the element pattern, while the magnetic field concentrates on the ITO pattern, as shown in Figure 4a–d. In addition, the distribution of the electric field and magnetic field between ITO patterns at 14 GHz is stronger than that at 6.5 GHz. The power loss density distributions, as shown in Figure 4e,f, which are similar to that of the electric field distributions, indicate that the power loss induced by the FTBMA can be attributed to the electric field coupling caused by resistance loss (ohmic dissipation). It can be observed that the direction of the current flow on bottom ground plane is opposite to that of the top ITO metasurface at 6.5 GHz from Figure 4g,i. Such anti-parallel current flows imply the occurrence of magnetic resonances between the top and bottom layer. However, the working mechanism for higher frequencies is quite different. As shown in Figure 4h,j, the direction of the current flow between the top and bottom layer is parallel at 14 GHz, which indicates electric resonances. It can be concluded that the electric and magnetic excitations occur simultaneously, resulting in the strong broadband absorption.

**Figure 5:**
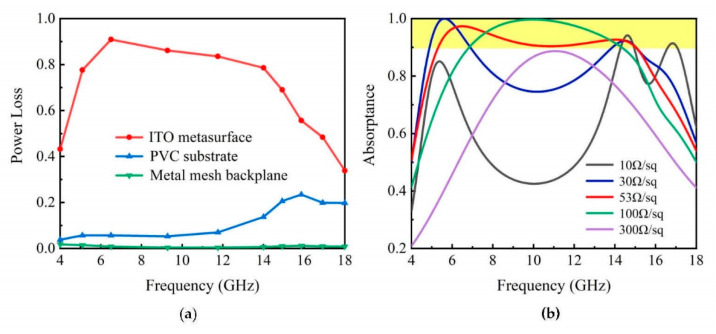
(**a**)The simulated power loss of the constitutive components in the FTBMA. (**b**) Simulated absorptance of the proposed structure for various upper-layer surface resistance (R_1_) values.

Then, the simulated power loss of the constitutive component in the FTBMA were calculated and given in Figure 5a. From the calculated results, it is obvious that the ITO metasurface on the top layer contributes the major power loss within the working bandwidth in the FTBMA, while the PVC substrate takes effect only for the high frequency band of 12–18 GHz, and there is almost no power loss in the Cu-metal-mesh backplane. Therefore, the ohmic loss from the patterned ITO metasurface makes a significant contribution to the excellent absorption performance of the FTBMA. The influence of various sheet resistances (R_1_) of the ITO metasurface on the absorption performance of the absorber was further explored, as shown in Figure 5b. It can be observed that, as the upper-layer resistance increases, the absorption strength of the FTBMA increases as a whole. However, the absorption peaks move towards each other and eventually merge into one absorption peak, resulting in a narrower working bandwidth when it increases to a certain extent. After genetic algorithm optimization, an optimum sheet resistance value (R_1_ = 53 Ω/sq) can be obtained for the widest absorption bandwidth (above 90%) of the FTBMA.

**Figure 6:**
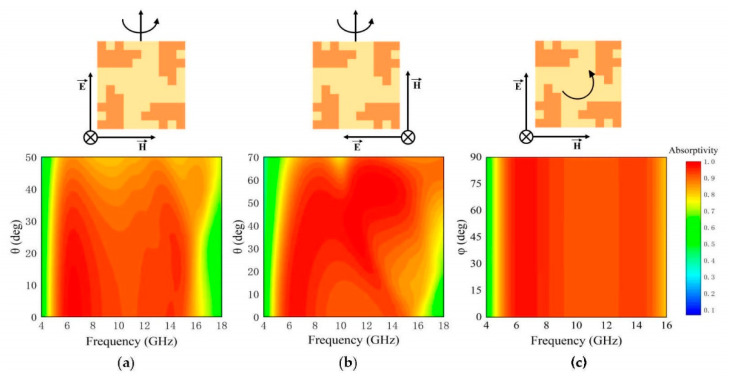
Simulated absorptivity as a function of incidence angle, θ, for (**a**) TE and (**b**) TM polarizations. The polarization orientation with respect to the surface in each case is shown schematically above the plot. (**c**) Simulated absorptivity as a function of polarization angle (φ).

It is necessary that absorbers have a good angular stability in many practical applications. Simulated absorptivity spectra under different oblique incidences of irradiation for TE and TM modes are illustrated in Figure 6a,b. Under TE mode irradiation, the absorption is maintained above 90% at incident angles from 0 to 30°, while the absorption band remains nearly unchanged. When the incident angle exceeds 30°, the absorption slightly decreases. By comparison, it can be observed that the absorptivity is higher at different incident angles under the TM mode, which indicates the incident angle stability is better for the TM mode. Moreover, it is clear that the absorption band slowly and gradually shifts to a higher frequency range under the TM mode. Overall, the absorptivity of the metamaterial absorber can be maintained above 85% across the working bandwidth at incident angles within 45° and 70° for TE mode and TM mode, respectively. These results indicate that the FTBMA features a reasonably stable angular performance. Moreover, the FTBMA has the advantage of polarization independence, thanks to its four-fold rotational symmetry, as shown in Figure 6c.

**Figure 7:**
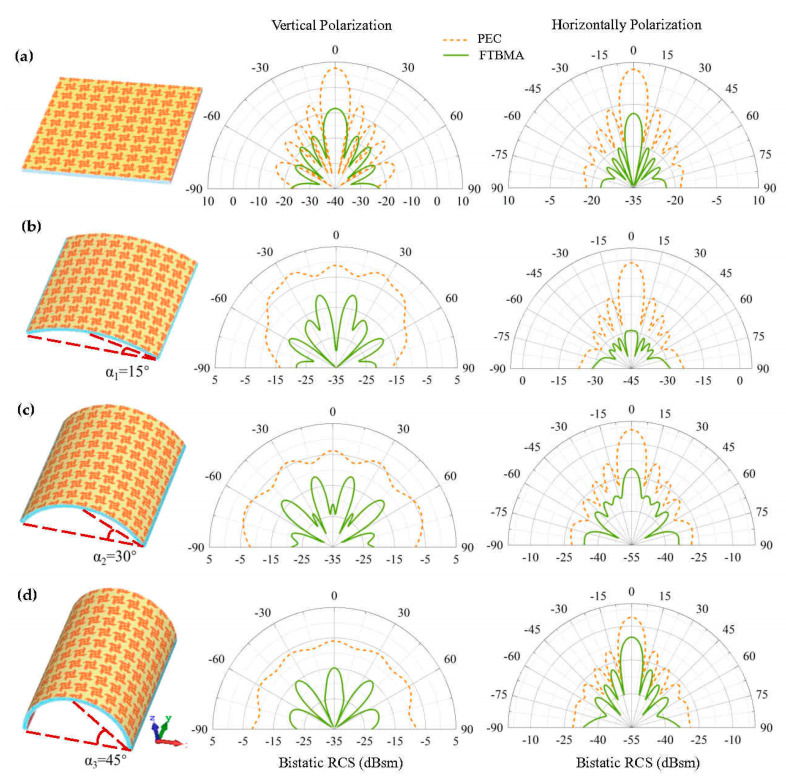
Schematic diagram of FTBMA (**left**) and bistatic RCS comparison of the metal plate and the FTBMA for vertical (**center**) and horizontal (**right**) polarized incident waves at 10 GHz: (**a**) both flat structures; (**b**–**d**) both curved structures with bending angles of α_1_ = 15°, α_2_ = 30°, α_3_ = 45°, respectively.

In general, the FTBMA with conformal profiles is more attractive in practical applications, because it has a powerful ability to be flexibly wrapped onto non-planar or even arbitrary shapes. To confirm the absorption properties of the FTBMA in the conformal case, a bistatic radar cross section (RCS) comparison of the metal plate and the FTBMA for vertical and horizontal polarized incident waves at 10 GHz are performed when it is bent into different bending angles of α1 = 15°, α_2_ = 30° and α_3_ = 45°, as shown in Figure 7. Note that the FTBMA composed of 10 × 10 unit cells is designed, and for comparison, a metal plate with the same size as that of FTBMA is also designed. The incident angle is θ_i_ = 0°, φ_i_ = 0° for vertically polarized and θ_i_ = 0°, φ_i_ = 90° for horizontally polarized incident. As shown in Figure 7a, the flat FTBMA exhibits good RCS reduction performance. It is also obvious that all the three curved FTBMAs can reduce the RCS by more than 10 dB compared to that of the metal plate with the same bending angle for both vertical and horizontal polarized incident waves in Figure 7b–d. The directivity of the scattered beam and RCS reduction decreases as the bending angle increases, which can be easily understood since a smaller effective aperture size is achieved for a larger bending angle. It is worth mentioning that the recently developed diffusion-like metasurfaces have capabilities for backward RCS reduction that are similar to those of the FTBMA in this work. However, the underlying functional mechanisms are quite different. The diffusion-like metasurfaces scatter the incident electromagnetic energy into different directions by creating a diffuse reflection effect through the disorder of the surface phase, resulting in the reduction in RCS peak [39,43]. However, they inherently enhance the magnitude of the side-lobes of the RCS [34,39]. Unlike the diffusion-like metasurfaces, the FTBMA converts the incident electromagnetic energy into heat energy through the strong resonance of the unit cells, which causes the RCS reduction. By contrast, the FTBMA has the advantage in that it allows RCS reduction for every reflection angle whether it is bent or not, as confirmed in Figure 7. These results show that the proposed FTBMA has a significant absorbing performance whether it is flat or curved.

## 4. Experiment and Discussion

**Figure 8:**
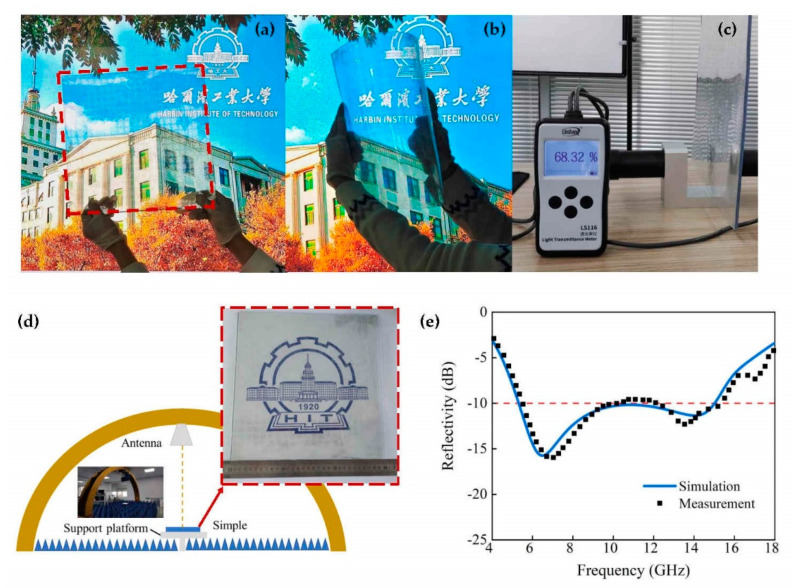
Photos of the fabricated (**a**) FTBMA and (**b**) bent FTBMA with great flexibility. (**c**) Transparency measurement of FTBMA. (**d**) Schematics of setups for measurement of reflectance. Inset: photograph of the fabricated FTBMA. (**e**) Comparison of measured and simulated reflectivity for FTBMA.

To experimentally verify the absorption performance of the FTBMA, the absorber sample consisting of 21 × 21 unit cells (294 × 294 mm^2^ surface area) was fabricated, as shown in Figure 8a,b and the inset of Figure 8d. The upper ITO film with a sheet resistance of 53 Ω/sq was deposited on a PET substrate by magnetron sputtering. Then, the resistive ITO film was patterned by laser ablation. The backplane Cu metal mesh with extremely low sheet resistance down to 1 Ω/sq and high transmittance of 86.5% was fabricated by selective electroplating of Cu in roll-to-roll imprinted microgrooves on the PET substrate [44]. Finally, the upper and lower layers were stretched and taped over a 4-mm, flexible and transparent intermediate PVC layer. An ultrathin layer of optically clear adhesive was used for interlayer adhesion with excellent light transmittance performance. Moreover, by using soft substrates like PET and PVC, the proposed FTBMA is flexible, as shown in the inset of Figure 8b.

A comparison of the measured and simulated reflectivity of the FTBMA is presented in Figure 8e. It can be seen that the experimental and simulation results are in good agreement. The minor deviation between the simulated and measured absorption bandwidths is primarily due to the fabrication and assembly tolerances, measurement errors and the variance of permittivity of the substrates. Moreover, the average transmittance of FTBMA in the visible band is approximately 68.3%, which was measured by using the optical transmittance tester LS116, as shown in Figure 8c.

## 5. Conclusions

In this work, a conformal metamaterial absorber with simultaneous optical transparency and broadband absorption is proposed by topology optimization combined with a GA. The proposed FTBMA offers microwave absorption at above 90% over a fractional bandwidth of 95.6% (5.3–15 GHz), and its thickness at the lowest cutoff frequency is 0.109λ_L_. Compared with previously reported transparent absorbers, our design exhibits significant broadband absorption, a low profile and flexible characteristics, which indicate that the FTBMA has the best overall performance, as summarized in Table 1. It is foreseeable that the FTBMA has potential utilizations in practical applications due to its flexibility, low profile, polarization insensitivity, oblique incidence stability and high optical transmission as well as its broadband absorption characteristics. Moreover, the proposed design method of topology optimization can be extended to design the absorber quickly and efficiently, according to specific engineering requirements.

The meanings of various acronyms used in this paper are listed in Table 2.

## Figures and Tables

**Table 1:** Comparison with other transparent broadband absorbers.

Absorber Structure	Relative Thickness (λ_L_) ^1^	90% Absorption Bandwidth (GHz)	*FBW*	Flexible	FoM
[35]	0.145	8.3–14.7	70.8%	NO	4.425
[17]	0.142	5.8–12.2	71.1%	YES	5.007
[7]	0.137	6–15.6	88.9%	YES	6.489
[45]	0.178	26.5–40	40.6%	YES	2.281
This work	0.109	5.3–15	95.6%	YES	8.771

^1^ λ^L^ is the wavelength of the lowest cutoff frequency.

**Table 2:** Acronyms used in this paper.

Acronym	Meaning/Full Form
FTBMA	Flexible Transparent Broadband Metamaterial Absorber
GA	Genetic Algorithm
*FBW*	Fractional Bandwidth
FoM	Figure of Merit
RFID	Radio Frequency Identification
ETC	Electronic Toll Collection
ITO	Indium Tin Oxid
PMMA	Polymethyl Methacrylate
PVC	Polyvinyl Chloride
PET	Polyethylene Terephthalate
CST MWS	Computer Simulation Technology Microwave Studio
TE	Transverse Electric
TM	Transverse Magnetic
PBCs	Periodic Boundary Conditions
